# Mental health challenges and substance use in student-athletes: prevalence and impact

**DOI:** 10.3389/fspor.2025.1527793

**Published:** 2025-03-14

**Authors:** Erin M. Moore, Maylasia A. Simmons, Julianna Threatt

**Affiliations:** Department of Kinesiology, University of Virginia, Charlottesville, VA, United States

**Keywords:** anxiety, depression, alcohol, cannabis, college athletes

## Abstract

**Background:**

Mental health significantly impacts athletes’ daily functioning and performance. Some coping techniques, such as substance abuse, can lead to addictive behaviors detrimental to sport participation.

**Purpose:**

This study aimed to identify the prevalence of anxiety, depression, and substance use in varsity student-athletes, examine their associations, and determine if academic and athletic factors (e.g., competition level, current sport season) are linked to these issues.

**Methods:**

An anonymous survey assessed mental health (anxiety and depression) and substance use in varsity athletes aged 18–25 participating in organized sports (high school, Junior College, NAIA, and NCAA Divisions I, II, III) and enrolled in academic classes. Four validated surveys were used: GAD-7 for anxiety, PHQ-9 for depression, AUDIT for alcohol use, and TAPS for substance use.

**Results:**

Sixty-two participants (19.87 ± 1.47 years; males: 30.6%, females: 67.7%) completed the study. Most participants (45.2%) were in-season, and 54.8% competed at the NCAA Division III level. Elevated levels of anxiety (64.5%), depression (62.9%), and substance use (alcohol: 59.7%; other substances: 49.18%) were reported. Only two participants reported illicit drug use (mushrooms). Significant associations were found between mental health issues, substance use, and athletic factors (competition level, sport season, academic year, sex).

**Conclusion:**

This study highlights the high prevalence of anxiety, depression, and substance use among student-athletes, particularly at the Division III level. These issues affect both academic and athletic performance. Clinicians should focus on early screening and be competent in recognizing and addressing mental health problems in student-athletes to make effective referrals.

## Introduction

Mental health has been brought to the forefront of the sports world in recent years with a focus on anxiety and depression. With prominent sports stars and other organizations addressing mental health in athletes, expectations and the conversation around overall health care has changed to encompass not only physical health, but mental health. Mental health includes the emotional, psychological, and social well-being of individuals and it affects the way that they think and act (1). Student-athletes experience multifaceted and simultaneous stressors including academics, sports, personal and social lives, social media, and now Name, Image and Likeness (NIL) agreements. These increased stressors make them more susceptible to developing mental health issues such as anxiety and depression (1, 2). The Diagnostic and Statistical Manual of Mental Health Disorders 5th edition (DSM-5) defines anxiety as the excessive worry or apprehensive expectation occurring more days than not for at least six months, about numerous events or activities (including work, school, etc.) where the individual finds it difficult to control the worry, and the anxiety is associated with three or more of the following six symptoms: Restlessness or feeling keyed up or on edge, being easily fatigued, difficulty concentration or mind going blank, irritability, muscle tension, and sleep disturbances (3). The DSM-5 defines major depressive disorder with discrete episodes of at least 2 weeks’ duration (most episodes last longer) involving clear-cut changes in affect, cognition, and neurovegetative functions and inter-episodes remissions (3).

Previous research from the National Collegiate Athletic Association (NCAA) in 2012, demonstrated elite-level athletes have increased mental health issues (31% of males, 48% of females), including anxiety and depression, partly due to the demands of being student-athletes (2). As of 2022, these rates remain elevated by 1.5–2 times higher than pre-Covid-19 pandemic measurements (4). Literature shows that student-athletes have a 71% likelihood of dropping out during their first year of college compared to non-athletes (5). An athlete's personal perception on mental health is a major determining factor in whether they will seek out treatment when needed (6). Although student-athletes admit to having mental health struggles, some literature found only about 10% of those that admit to struggling will seek help or treatment for their problems (7, 8). While the NCAA polled athletes (55% in men's sports, 47% in women's sports) who felt their mental health was *a priori*ty to the athletics department, 59% of men's sports and 50% of women's sports felt their coach took their mental health concerns seriously (9). Additionally, 58% of men's sports and 65% of women's sports felt teammates took mental health concerns seriously (9). Finally, 56% of both men's and women's sports participants knew how to help a teammate experiencing a mental health issue (9). While these numbers may not add up, this could be related to the ongoing stigma surrounding mental health. There is a clear and pervasive stigma that surrounds mental health across many populations and cultures, particularly in athletics. It is important to note that there is both a public stigma and self-stigma within the discussion of mental health in college athletics. Public stigma refers to the negative stereotypes that people associate with mental health issues, while self-stigma is the internalized thoughts and impacts of these public stigmas (10). Athletes often view seeking help for mental health issues as weak and symbolizing instability within themselves compared to the recent positive changes in public stigma (5). This is especially true for male athletes, which is one reason they report fewer anxiety and depression symptoms compared to female athletes (10). Males want to maintain the masculinity norms that are present (public or self-identified), creating another barrier for men who struggle with deciding whether to seek treatment (6). Leaving mental health issues untreated can lead to poor coping mechanisms and impact their athletic performance as well as their personal life (6).

When addressing stressors (internal and external) in sport, utilizing the stress and coping theory form Lazarus and Folkman (11). This theory addresses an individual's reaction to stress is influenced by their own perception of stress and the available resources regarding to cope with stressors. Utilizing this framework can help examine and understand how athletes may perceive their stresses, subsequent mental health conditions (anxiety and depression) and chosen coping methods that can have a large impact on health and performance (11, 12). Coping methods (maladaptive or adaptive) which can impacted be either personality factors, personal resilience to stress and individual flexibility within coping strategies (11, 12). The importance of adaptive coping strategies allows for the effective coping to either increase performance and mental health or decrease negative effects on performance and mental health (11, 12). One poor coping mechanism that impacts athletic performance, eligibility and their personal life includes substance use (alcohol, tobacco, prescription medications, and recreational/illicit drugs). Substance use is defined as the use of certain substances such as alcohol, tobacco, and other substances/entities that can be inhaled, injected, or consumed into the body with the possibility/risk of gaining dependence (13). Alcohol is one of the most predominant substances used and abused with ∼30% of student-athletes experiencing blackouts (14). One form of abuse includes binge drinking. Defined specifically as alcohol consumption that raises the blood alcohol concentration to 0.08% or higher (above the legal limit) in one sitting/occasion (15). This is more commonly defined as 4 or more drinks in one sitting for females, and 5 or more drinks in one sitting for males (15). The NCAA noted across all 3 divisions, that student-athletes typically binge drink, with ∼30% of females consuming 4 or more drinks in one sitting and ∼40% of males having 5 or more drinks (14). With increased uses of drinking and other substances, student-athletes are at an increased risk of substance abuse/disorder.

Substance use disorder (SUD) is a treatable mental disorder that affects a person's brain and behavior, leading to their inability to control their use of substances like legal or illegal drugs, alcohol, or medications (13). The DSM-5 defines SUD as a cluster of cognitive, behavioral, and physiological symptoms indicating that the individual contuse using the substance despite significant substance-related problems (3). Addiction is the most severe form of SUD with 46.3 million people 12 years or older (16.5% of the population) meeting the DSM-5 criteria for SUD in 2021 with the highest percentage being among young adults aged 18–25 (13, 16). While those numbers sound troubling, what is even more concerning is that 94% of people did not receive any treatment due to the belief they did not need any help (16). Since the majority of the population suffering from SUD falls within the ages of 18–25, SUD and lack of treatment can be observed in the high school and collegiate athletic populations. In United States high schools, 47.4% of students reported using alcohol, 27.8% reported using marijuana, and 12.2% reported using opioids (13, 16). Consequences for student-athletes could be negatively impactful to sport participation, (suspension from the sport or NCAA) possible loss of a scholarship, and potentially now loss of NIL deals. Student-athletes tend to use these substances to help with depression and anxiety, however, a compounding factor of substance use, includes the exacerbation of mental illness symptoms such as depression, anxiety, attention-deficit hyperactivity disorder, post-traumatic stress disorder, obsessive-compulsive disorder, and schizophrenia (3, 13). Roughly 14% of young adults present with both SUD and mental illness (16). Regarding the treatment of substance use, the general population has support such as medical insurance, availability, and access when seeking help. Student-athletes have even more accessible resources for healthcare and assistance with substance use issues at any stage of their treatment. However, similar to the general population, most individuals do not seek treatment. For those who do seek treatment or show obvious signs, healthcare workers can use screening tools to identify individuals who may need help or to assess problematic usage.

Mental health and coping strategies play a significant role in an athlete's daily functioning, overall health, and sport performance. Despite some advancements and increased awareness of the importance of mental health, there remains substantial barriers to assessing and addressing mental health issues in student-athletes, especially after a global pandemic, increases in social medica and the new NIL requirements in recent years (2). Therefore, the purpose of this study was to identify the prevalence of anxiety, depression, and substance use in varsity student-athletes (age 18–25). Additionally, the study aimed to examine whether anxiety and/or depression are associated with substance use (AUDIT and/or TAPS scores). Finally, it sought to determine if academic and athletic factors (e.g., competition level, current sport season, etc.) are associated with anxiety, depression and/or substance use.

## Materials and methods

### Experimental design and participants

This study utilized an anonymous survey to examine mental health (anxiety and depression) and substance use prevalence in varsity athletes ranging between 18 and 25 years old participating in varsity level organized sports (high school [HS], Junior College [JUCO], National Association of Intercollegiate Athletics [NAIA], and National Collegiate Athletic Association Divisions I, II, and III [NCAA D1, D2, D3] and enrollment in academic classes/school. A total of 62 participants (19.87 ± 1.47 years, males: *n* = 19 30.6%, females: *n* = 42, 67.7%, other: *n* = 1, 1.6%) completed the study in its entirety. Exclusion criteria include any student-athlete younger than 18 years (no minors) and any student-athlete over 25. Any student-athlete not actively participating at varsity level during the timeframe of the survey (i.e., junior varsity and recreational athletes).

### Instruments/protocols

This study utilized four reliable and validated surveys to assess two mental health conditions of anxiety (GAD-7) and depression (PHQ-9) and the usage of substances including alcohol (AUDIT) and all substances (TAPS).

#### Generalized anxiety disorder-7 (GAD-7)

The Generalized Anxiety Disorder-7 (GAD-7) is a 7-item questionnaire that quantifies a subject's levels of anxiety. It has been widely used in research and clinically through healthcare professionals. It is a short and easy questionnaire to take, making it versatile for different settings and populations. Subjects are instructed to respond to 7 questions based on how they have been feeling the past 2 weeks. Subjects may answer “not at all”, “several days”, “more than half the days”, or “nearly every day” for each question. The responses are respectively scored 0–3 on a 4-point Likert scale with 0 being “not at all” and 3 being “nearly every day”. Upon completion, all the columns are added up to produce a total score ranging from 0 to 21. The scores are grouped into categories of minimal anxiety, mild anxiety, moderate anxiety, and severe anxiety (7). A score of 10 or above is the threshold for moderate anxiety and it is also the point for when a referral is needed for further follow-up. For this study, any scores 5 and above (mild to severe) will be assessed as elevated anxiety prevalence. The GAD-7 is a validated scale with a sensitivity of 89% and specificity of 82% when using the cut-off score of 10 (8). It also has excellent internal consistency and test-retest reliability with a Cronbach Alpha of 0.92 along with an intraclass correlation coefficient of 0.83 (8).

#### Patient health questionnaire-9 (PHQ-9)

The patient health questionnaire (PHQ-9) is a 9-item diagnostic tool participants completed to self-report depression symptoms. Healthcare professionals can use the PHQ-9 to screen subjects when diagnosing major depressive disorder. Subjects respond to each of the questions based on what they have been feeling and/or experiencing over the past two weeks. The symptoms are rated based on the frequency that the participant experiences them. The responses to each question were divided into never, several days, more than half of the time, and almost every day. If the subject responded they had any of the problems, a tenth question would warrant a response about how difficult the problems make it for the subject to carry out their daily activities. Scoring ranged from 0 to 27 with scores of 5–9 representing mild depression, 10–14 representing moderate depression, and 15–19 representing moderately severe depression, and 20–27 representing severe depression symptoms (8). A cut-off score of 10 is also indicative of having a depressive disorder for the general population (8). For this study, any scores above 5 or above (mild to severe depression) will be assessed as elevated scores of depression prevalence. The PHQ-9 has a sensitivity of 88% and a specificity of 88% (8). Cronbach's alpha value of these 9 items was 0.88, which was larger than 0.70 and good test-retest reliability (intraclass correlation coefficient ≥0.80) (8, 17). The PHQ-9 should not be the sole determining factor in whether or not an individual may have depression, but instead it should be used as a screening tool to help rule out depression. Due to the sensitivity of mental health and the questions asked in the survey, participants could withdraw at any point during the survey and National Mental Health resources were provided on the consent page and at the end of the survey if needed.

#### Alcohol use disorders identification test (audit)

The AUDIT is the gold standard tool for determining the severity of alcohol use and should be used for an individual expressing a concern with their drinking habits (18). The AUDIT determines the severity of an individual's drinking habits based on the answers selected to 10 questions that correspond to a numeric value (18). All the answers are added together to a final score (18). A score of 0 indicates an abstainer with no issues from alcohol, a score of 1–7 suggests low-risk consumption, 8–14 is hazardous or harmful consumption, and a score of 15 + indicates a high likelihood of alcohol dependence (18). For this study, any scores 1 and above (low risk to high likelihood of alcohol dependence) will be assessed as elevated scores of alcohol consumption. This tool has a sensitivity of 0.98 and specificity of 0.94 for scores greater than 8 (18). For patients under the age of 18, a better tool to use is the Alcohol Screening and Brief Intervention for Youth (9–18 years old) (19).

#### Tobacco, alcohol, prescription medication, and other substance use (TAPS)

The TAPS is the gold standard tool for assessing substance use risk for the adult population (ages 18 and older). The first component (TAPS-1) asks about history of substance use in the past 12 months, if those questions are answered yes then the second component (TAPS-2) will be prompted which asks about the use in the past 3 months (20). Unlike the AUDIT, TAPS does not use an overall numerical scale to determine the severity, instead TAPS uses the answers based on per substance use to generate risk level per substances selected. By answering yes, an individual receives a 1 and then the total score for that substance is calculated (20). Risk categories include No Use in the past 3 months (0 score), Problem Use (1 score) and Higher Risk (2 + score) (20). For this study, a score of 1 or higher (problem use to higher risk) will be assessed as elevated scores of substance use. TAPS is the preferred method compared to other tools due to its high sensitivity and specificity with tobacco (sensitivity 0.92, specificity 0.87), alcohol (sensitivity 0.77, specificity 0.77), and commonly used classes of illicit drugs (sensitivity ranging from 0.73 to 0.79, specificity ranging from 0.93 to 1.0) (21). For adolescent patients aged 12–21 can use the CRAFFT screening tool (19). When using the TAPS as part of diagnostic criterion for DSM-5 SUD, it is recommended to use a cutoff score of 2 or higher (20). This cutoff provides adequate sensitivity (>70%) only for tobacco, alcohol, and marijuana (20).

### Procedures

An anonymous survey was created through Qualtrics Experience and was approved by the University's IRB (SBS#6190). The survey was sent out in September 2023, attached to an IRB-approved flyer on multiple social media platforms including University athletic programs, University Master of Athletic Training programs platforms, and Morgan's Message platforms where the public were able to view the flyer and participate accordingly. The consent information was provided on the survey's first page, that participants voluntarily viewed via a QR Code/link. Participants could click no and exit out of the survey at any time. The survey ended in March 2024. The survey ranged from 5 to 15 min to take depending on the participant's responses. The survey consisted of demographic questions including sex, age, race, sport, stage of sport season, division/competition level, academic year, and athletic year, the GAD-7, PHQ-9, AUDIT, and TAPS. Due to the nature of the questions in this survey that are associated with mental health and substance abuse, at the end of the survey, the following statement and resources were provided.

“In the event any of the previous questions caused you duress or made you feel uncomfortable, please reach out to your health care provider or google “find mental health support near me”. Other resources to reach out to include NIH- with a live chat feature: https://www.nimh.nih.gov/health/find-help; National Mental Health Hotline 866-903-3787; mental health.gov; and call Suicide Hotline: 988.”

### Statistical analysis

IBM SPSS statistical Software (version 29; SPSS Inc., Armonk, NY) and an alpha error (*α*) of ≤0.05 were used for all analyses to determine statistical significance. An *a priori* power analysis using G*Power software (version 3.1.97, Heinrich Heine University, Dusseldorf, Germany) calculated power using chi-square analysis for PHQ-9, GAD-7, AUDIT and TAPS, with an alpha of 0.05. With an effect size of 0.6, 52 subjects would allow for full saturation, with power at 0.952. Descriptive statistics for all dependent variables were calculated with frequencies and proportions with 95% confidence intervals for all categorical variables (GAD-7, PHQ-9, AUDIT, TAPS). Crosstabulations and chi-square analysis were used to examine “at risk” for anxiety, depression, alcohol problems, substance use and secondary measures (sports seasons, competition level, academic status, and athletic status). Pearson's correlations were used to examine relationships between anxiety, depression, substance use, and other academic and athletic factors.

### Results

Of the 106 participants that started the survey, 44 individuals were removed due to incomplete data (*n* = 36) and not meeting inclusion criteria (*n* = 8) resulting in a 58.5% completion rate yielding 62 participants. Self-reported ethnicity demonstrated participants identified as 77.4% (*n* = 48) Caucasian/White, 8.1% (*n* = 5) African American/Black, 8.1% (*n* = 5) Multiple ethnic/other), and 6.5% (*n* = 4) Hispanic. Academic and collegiate statuses by year, competitive season and competition level are presented in Table 1. The majority of participants (45.2%, *n* = 28) completed the survey while they were in-season of their respective sport. Approximately 54.8% (*n* = 34) of respondents reported competing at the NCAA Division 3 level while only 22.6% (*n* = 14) reported participating in NCAA Division 1 athletics (see Table 1*).* Many participants demonstrated elevated levels of anxiety (64.5%, *n* = 40) depression (62.9%, *n* = 39), and increased use of substances (AUDIT: 74.2%, *n* = 49; TAPS: 69.3%, *n* = 43) in varsity student-athletes (see Table 2). Of note, only 2 participants reported drug use, both used mushrooms as their drug of choice. Sex differences assessing risk or no risk for the four screening tools are presented in Table 3. Overall, significant relationships and associations were found between anxiety, depression, substance use and athletic factors (competition level, current sport season, academic year, sex, etc). Pearson correlations were calculated to examine the relationships between the anxiety (GAD-7) depression risk (PHQ-9), alcohol use (AUDIT), substance use (TAPS), DSM-5 SUD status, demographics (age, sex, ethnicity, academic year) and athletic status (athletic year, sport season) (see Table 4). When examining sex differences between males and females, only TAPS scores indicated a significant difference [*χ*^2^ (7) = 15.93, *p*-value = 0.03].

**Table 1:** Demographic data on academic year, athletic year, competitive season, and competition level (*n* *=* 62).

Academic year	%	*N*
Freshman	19.4%	12
Sophomore	27.4%	17
Junior	21%	13
Senior	29%	18
Graduate	3.2%	2
Athletic year	%	*N*
Freshman	21%	13
Redshirt freshman	6.5%	4
Sophomore	17.7%	11
Redshirt sophomore	8.1%	5
Junior	11.3%	7
Redshirt junior	4.8%	3
Senior	29%	18
Redshirt senior	1.6%	1
Competitive season	%	*N*
Pre-season	17.7%	11
In-season	45.2%	28
Post-season competitive play	3.2%	2
Post-season non-competition requirements and no formal practice/training	14.5%	9
Out of season—includes practices and training	19.4%	12
Competition Level	%	*N*
NCAA division 1	22.6%	14
NCAA division 2	6.5%	4
NCAA division 3	54.8%	34
Varsity high school	12.9%	8
JUCO	1.6%	1
NAIA	1.6%	1

Values are presented in number (*N*) and percentage (%). NCAA, National Collegiate Athletic Association; JUCO, Junior College; NAIA, National Association of Intercollegiate Athletics.

**Table 2:** Self-Reported anxiety, depression and substance Use scores from GAD-7, PHQ-9, AUDIT and TAPS (*n* *=* 62).

Generalized anxiety disorder-7	%	*N*
Minimal anxiety (0–4 points)	35.5	22
Mild anxiety (5–9 points)	38.7	24
Moderate anxiety (10–14)	14.5	9
Severe anxiety (15–21)	11.3	7
Patient health questionnaire-9	%	*N*
Minimal depression (0–4 points)	37.1%	23
Mild depression (5–9 points)	48.4%	30
Moderate depression (10–14 points)	11.3%	7
Moderately severe depression (15–19 points)	3.2%	2
Severe depression (20–27)	0%	0
Alcohol use disorders identification test	%	*N*
No risk	25.8%	17
Low risk	53%	35
Harmful	13.6%	9
High risk of dependence	7.6%	5
Tobacco, alcohol, prescription medications and other substance use	%	*N*
0	29%	18
1	21%	13
2	17.7%	11
3	11.3	7
4	12.9%	8
5	3.2%	2
6	1.6%	1
7	1.6%	1

Values are presented in number (*N*) and percentage (%).

**Table 3:** Risk to depression interactions with competition level, season, and sex (*n* = 62).

GAD-7	No risk to anxiety		Anxiety risk		*p*-value
Sex	*N*	%	*N*	%	0.35
Female	13	31%	29	69%	
Male	9	47.4%	10	52.6%	
PHQ-9	No risk to depression		Depression risk		*p*-value
Sex	*N*	%	*N*	%	0.78
Female	15	35.7%	27	64.3	
Male	8	13.1%	11	18%	
AUDIT	No risk		Alcohol dependency risk		*p*-value
Sex	*N*	%	*N*	%	0.07
Female	2	4.8%	40	95.5%	
Male	0	0%	19	100%	
TAPS	No risk		Problem use/high risk		*p*-value
Sex	*N*	%	*N*	%	0.03*
Female	12	29.3%	29	70.7%	
Male	6	31.6%	13	68.4%	

Values are presented in number (*N*) and percentage (%). GAD-7, generalized anxiety disorder-7; PHQ-9, patient health questionnaire-9; AUDIT, alcohol use disorders identification test; TAPS, tobacco, alcohol, prescription medications and other substance use.

* *a priori p*-value <0.05 considered significant.

**Table 4:** Pearson correlations examining relationships for mental health, substance use, demographics, and athletic status (*n* *=* 62).

Variables	Correlation	*P*-value
Generalized anxiety disorder-7		
PHQ-9	*r* (60) = .690	<0.001
AUDIT	*r* (60) = .270	0.03
TAPS	*r* (59) = .277	0.03
DSM-5 SUD	*r* (59) = .283	0.03
Athletic year	*r* (60) = .260	0.04
Sport season	*r* (60) = −.359	0.004
Patient health questionnaire-9		
AUDIT	*r* (60) = .348	0.01
TAPS	*r* (59) = .364	0.004
DSM-5 SUD	*r* (59) = .333	0.01
Ethnicity	*r* (60) = −.273	0.03
Sport season	*r* (60) = −.335	0.01
Alcohol use disorders identification test		
TAPS	*r* (59) = .726	<0.001
DSM-5 SUD	*r* (59) = .575	<0.001
Age	*r* (60) = .291	0.02
Academic year	*r* (60) = .270	0.04
Athletic year	*r* (60) = .278	0.03
Tobacco, alcohol, prescription medications and other substance use		
DSM-5 SUD	*r* (59) = .816	<0.001
Age	*r* (59) = .387	0.002
Academic year	*r* (59) = .303	0.02
Athletic year	*r* (59) = .340	0.01
Sex	*r* (59) = −.278	0.03
DSM-5 substance use disorder		
Age	*r* (59) = .276	0.03
Sex	*r* (59) = −.344	0.007

Values are presented in correlation and *p*-values. Only results with an *a priori p*-value ≤0.05 considered significant were reported, all other variables were not significant and therefore not presented or listed. GAD-7, generalized anxiety disorder-7; PHQ-9, patient health questionnaire-9; AUDIT, alcohol use disorders identification test; TAPS, tobacco, alcohol, prescription medications and other substance use; DSM-5, diagnostic and statistical manual of mental health disorders -5th edition; SUD, substance use disorder.

### Mental health

#### Anxiety

Regarding anxiety, a total of 64.5% (*n* = 40) participants reported having symptoms of anxiety. Of those individuals, 9 individuals said that these symptoms make it “very difficult” to go about their everyday life and 2 individuals reported that it was “extremely difficult” to do so. Scores of the GAD-7 ranged from 0 to 21 with the mean score of all participants being 6.84 ± 4.94, demonstrating mild anxiety levels. Of the 62 participants, 16 (25.8%) met the threshold score of ≥10, identifying generalized anxiety disorder. Frequencies related to the GAD-7 can be seen in Table 2. When observing anxiety levels (GAD-7) in relation to depression levels (PHQ-9), substance use (AUDIT, TAPS, and DSM-5 SUD), and competition level, participants showed increased scores (*see* Table 5). All other variables did not demonstrate significant changes.

**Table 5:** Compromised mental health, substance Use in relation to athletic descriptive factors in varsity athletes.

Anxiety: GAD-7	%, *N*	Chi-square	*p*-value
PHQ-9	51.61%, *n* = 32	*χ*^2^ (9) = 39.10	<0.001
AUDIT	64.52%, *n* = 40	χ^2^ (6) = 12.97	0.04
DSM-5 SUD	62.90%, *n* = 39	χ^2^ (15) = 22.53	<0.001
Competition level	64.52%, *n* = 40	χ^2^ (15) = 30.54	0.01
Depression: PHQ-9			
Sport season	64.52%, *n* = 40	χ^2^ (15) = 30.54	0.01
Substance use AUDIT			
TAPS	70.50%, *n* = 43	χ^2^ (14) = 40.31	<0.001
DSM-5 SUD	30.8%, *n* = 4	χ^2^ (4) = 26.45	<0.001
Substance use TAPS			
Athletic year	69.35%, *n* = 43	χ^2^ (49) = 82.82	0.002
Sex	M = 68.4%, *n* = 13,	χ^2^ (7) = 15.93	0.03
	F = 70.7% *n* = 29		

Athletes *n* = 62. Values are presented in number (*N*) and percentage %, Chi-square, and *p*-values. Only results with an *a priori p*-value ≤0.05 considered significant were reported, all other variables were not significant and therefore not presented or listed. GAD-7, generalized anxiety disorder-7; PHQ-9, patient health questionnaire-9; AUDIT, alcohol use disorders identification test; TAPS, tobacco, alcohol, prescription medications and other substance use; DSM-5, diagnostic and statistical manual of mental health disorders -5th edition; SUD, substance use disorder.

#### Depression

Examining depression, total scores of the PHQ-9 ranged from 0 to 22, out of a maximum score of 27. Results showed a total of 62.9% (*n* = 39) of the participants demonstrated at least a mild depression score. The average score across all participants was 7.5 ± 5.52 indicating an average risk of mild depression. A total of 14.5% (*n* = 9) individuals met the minimum score of ≥10 indicating a depressive disorder. A total of 11.3% (*n* = 7) individuals indicated having symptoms of suicidal ideation based off results from question 9 of the PHQ-9. Frequencies related to the PHQ-9 can be found in Table 2. When observing depression levels (PHQ-9) in relation to sport season, participants demonstrated increased scores while all other variables did not demonstrate significant changes (see Table 5).

#### Substance Use

Assessing alcohol consumption specifically, scores of the AUDIT ranged from 1 to 31, out of a maximum score of 40. Results demonstrated 13.6% (*n* = 9) of scores between 8 and 14, which is identified as hazardous or harmful consumption of alcohol and 7.6% (*n* = 5) scored 15 + and identified as high risk of dependence on alcohol, with an average score of 13.95 ± 5.75. One specific question within AUDIT asks about binge drinking behaviors and is identified as “how often do you have six or more drinks on one occasion?”. Results demonstrated 19.4% (*n* = 12) drank 6 or more drinks monthly, 17.7% (*n* = 11) drank 6 or more drinks weekly, and 8.1% (*n* = 5) drank 6 or more drinks daily or almost daily. Frequencies related to the AUDIT can be found in Table 2. Examining substance use, scores related to substance use [tobacco, alcohol, prescription medications and other substances (recreational and illicit drugs)] demonstrated an average of 1.82 ± 1.75 demonstrating problematic substance use. Frequencies related to TAPS can be found in Table 2. The majority of participants *(*48.3%, *n* = 30) scored at or above 2 on the TAPS, indicating a higher risk score. This score is part of the diagnostic criterion for the SUD from the DSM-5. When observing alcohol use specifically (AUDIT) in relation to TAPS, and DSM-5 SUD, participants showed increases scores while all other variables did not demonstrate significant changes. Additionally, when observing substance use (TAPS) in relation to Athletic Year, participants demonstrated increased scores. (*see* Table 5).

## Discussion

This study aimed to identify the prevalence of anxiety, depression, and substance use in varsity student-athletes (age 18–25). Although, in isolation, the GAD-7, PHQ-9, AUDIT, and TAPS are not used to diagnose anxiety and depression or substance use, they can identify the prevalence of these symptoms with cut off scores that would warrant follow-up with appropriate medical professionals. Of the participants in this study, almost 65% demonstrated a prevalence of mild to severe anxiety symptoms, 63% demonstrated a prevalence of mild to moderately severe depression symptoms, 74% demonstrated a prevalence of mild to high risk of dependence on alcohol, and almost 50% of participants had a score of 2 + on the TAPS, indicative of levels associated with a DSM-5 diagnosis of SUD.

### Anxiety

When examining previous literature regarding student-athletes and anxiety, these findings were not consistent. Existing literature found there was a higher prevalence of anxiety in student-athletes compared to depression in student-athletes (8). Results demonstrated that there was a relationship between the student-athletes’ sex and whether or not they met the threshold for anxiety. Females in this study also represented the majority of the individuals that demonstrated elevated levels of anxiety scores. These results align with previous literature where females experience anxiety and symptoms associated with anxiety in higher frequencies than their male counterparts (8, 22). These elevated scores are congruent with the NCAA's Student-Athlete Well-Being Study in May 2022, which demonstrated that anxiety rates remain 1.5–2 times higher than pre-Covid-19 pandemic measures (9). Some reasons for this anxiety is attributed to academic experiences, transferring, race and gender equity, and educational resources (4). Of note, more than half (55%) our participants participated in NCAA Division III athletics, which previous literature by Stokowski et al. (23), and Valster et al. (24), did not find concerning levels of anxiety which is not congruent with our literature. Stokowski et al. (23), surmised that Division III athletes and their experiences are not similar to those at the DI or DII and implementing similar systems may not be as impactful. However, our results as well as Bishop (25) found the opposite conclusion. Division III athletes have similar experiences and needs to those of DI and DII and desire more educational opportunities and resources that support mental health.

### Depression

On average, despite most participants in this study scoring in the mild depression range, ∼15% of the participants met the threshold for moderate depression and moderately severe depression. This is nearly equivalent to one in every three student-athletes reporting symptoms indicative of a depressive order. These findings are slightly higher than the 15.6% and 21% range that previous literature found (26, 27). Contrary to earlier research, our data did not find any significant relationships between sex and depression symptoms.

The final question on the PHQ-9 asks subjects about self-harm and suicidal ideation. This question is not scored any differently than the 8 other questions, however, there is some obvious weight associated with this question due to the sensitivity of self-harm and suicide. Our survey showed that 6 participants answered that they thought about harming themselves several days over the past 2-week span. One individual reported that the thought of harming themselves bothered them nearly every day. This finding is important to note as a recent 20 year analysis revealed that suicide is the second leading cause of death in NCAA student-athletes (28). Recent findings revealed D1 and D2 student-athletes have experienced higher rates of suicides compared to D3 student-athletes (28). While only a small proportion indicated symptoms of suicidal ideation based on question 9 of the PHQ-9, any signs and symptoms of depression (mild to severe) is problematic, and can those with signs and symptoms present with risks of leading to self-harming measures and suicide. A total of 15% of individuals met the threshold for depression according to our findings. The NCAA's Student-Athlete Well-Being Study also demonstrated depression rates remain 1.5–2 times higher than pre-Covid-19 pandemic measures while hopelessness levels decreased since the fall of 2021 (4). When considering the large Division III participation in our study, Stokowski et al. (23), did not find concerning levels of depression while Valster et al. (24), found less than 11% of DIII experienced depression and determined COVID-19 pandemic did not significantly impact mental health within Division III athletes. While Valster's findings are similar to our findings of moderate depression scores (∼11%), we also had some participants with moderately severe depression (∼3%) and a large percentage of mild depression scores (48%). Valster et al. (24), also found significant interactions between gender and depression in Division III athletes (*p* = 0.009), while our findings did not demonstrate any sex difference between males and females statistically. However, our participant data collection was over a shorter time period and contained a mix of collegiate levels (D1 and D3) when comparing to Valster et al.'s study of only D3 athletes. Congruent with previous literature, females did have a 3.5 times higher percentage of depression risk compared to males in this study even though we did not find statistical significance, it is important to understand females are continuing to present with increased depression and mental health risks. Healthcare professionals should screen elite female athletes to help identify those at risk of depression symptoms.

### Substance Use

This study identified those who participate in varsity sports as full-time student-athletes are at increased risk of substance use. Roughly 60% demonstrated harmful use of alcohol and 8% were at high risk of dependence while 70% had a score of 1 or more on the TAPS- suggesting either problematic use or higher risk of SUD. Examining substance use (Tobacco, Alcohol, illicit and recreational drugs) in NCAA and NAIA demonstrated similar results to this study, however, results were lower when compared to non-athletic college students (29–31). Moore and Abbe (29) examined substance use within the NAIA and the NCAA (30). They examined all three divisions regarding substance use and found alcohol use was lower in NAIA (∼50%) compared to NCAA athletes (77%), while student-athletes were lower compared to college students (∼80%) (29, 30). While our study did not have participants from the NAIA, our results were lower within NCAA D1 and D3 for alcohol use. While alcohol use is considered lower in student-athletes globally compared to non-athlete peers, binge drinking had higher prevalence rates within student-athletes (NAIA 19% and NCAA 42%) compared to non-athlete college students (3%) (29, 30). Previous studies showed that although student-athletes engage in binge drinking, which may account for higher scores on the TAPS (13). When we examine the questions specific to 6 or more drinks in on an occasion from the AUDIT, our results were lower than previous literature.

While we did not report on sport type, of note, team sports have more substance use among NAIA and NCAA student-athletes (29–31). Tobacco and Nicotine use is lower in student-athletes compared to non-athletes (30) ∼28% using some form of tobacco in 19 and older while 18% use was found in 18 year olds (31). The most common uses of tobacco included 8% using e-cigarettes/vaping and 13% using spit tobacco at least once in the last year with higher prevalence e-cigarette use in NAIA athletes (29). Marijuana uses also trends lower in student-athletes (25%) compared to non-athlete population (33%) (29, 30). Illicit drug use has been observed at ∼43% in college students. Specific to student-athletes rate are lower including ∼2% use of amphetamines, again lower than non-athletes (5%), while within student-athletes in the NCAA, ∼7% used Cocaine, 3.5% used Ecstasy/Molly, ∼3% used LSD, and less than 1% used Methamphetamines and heroin (30). Our study found only ∼3% of our participants used Mushrooms (microdose). Marijuana use was viewed at 21% of NAIA student-athletes, 35% NCAA compared to 39% college students (29–31). Marijuana use specifically in the NCAA Division III level demonstrated 32.6% which is slightly lower compared to all NCAA divisions (29). Medication use was observed at 11% in NCAA athletes, with 3% reported using without a prescription and 2% reported misusing narcotic pain medication (30). Overall, narcotic and pain medication use is similar to NAIA and NCAA athletes (29).

Substance use is typically used to mitigate stress or other symptoms related to mental health (e.g., anxiety and depression). Knettel (32) identified both academics and athletics are the top two greatest stressor in student-athletes lives. Knettel (32) identified reasons for substance use within this population and found they included pain, academic and athletic stressors, time management and emotional health as the top overall factors associated with binge drinking, cannabis use and other substances. Anxiety was a significant indicator of cannabis use and substance-risk behaviors and is understudied currently in literature (32). Those over the age of 21 and male student-athletes were more likely to report substance use and risk behaviors (32). Our study had an uneven distribution of females and males, and therefore not congruent with this previous literature. Substance use is present within college athletics and health care professionals need to be aware of use and abuse given the comorbidity with mental health as well as the NCAA rule changes around substance use. Screening tools and education on signs and symptoms is paramount for those working with collegiate athletes.

### Anxiety, depression and substance use

Additionally, our secondary aim was to examine the relationship between anxiety and depression associated with substance use (AUDIT and/or TAPS scores). There were many relationships and interactions between GAD-7 scores, PHQ-9 scores, AUDIT and TAPS scores. This is congruent from previous literature. Previous research has shown that comorbidities between anxiety, depression and substance use are well documented (3, 27, 33). These statistics are more concerning now that there are a number of newer factors affecting student-athletes in recent years. This includes the increased amount of media coverage they receive, increases in exposure due to social media, the incorporation of name, image, and likeness (NIL) deals at higher levels (28). Despite not having some of the same pressures or intensities of the same pressures as student-athletes at the Division I and Division II levels, anxiety and depression are still prevalent amongst Division III student-athletes. Finally, our study sought to determine if demographic, academic and athletic factors are associated with anxiety, depression and/or substance use. Overall, there were clinically meaningful results the showed increased levels of anxiety, depression and substance use. Elevated anxiety was linked to athletic year and sport season, which is congruent with previous literature (22, 24, 26). Depression scores correlated with ethnicity and sport season (5, 24, 26, 28). AUDIT scores were associated with age, sex, academic year and athletic year (29, 31, 32, 34, 35).

The NCAA does examine mental health in student-athletes and has examined relationships between mental health and substance use across division levels, which demonstrates the importance of mental health for student-athletes. Their research did not yield any statistically significant differences when looking at mental health and substance use across NCAA athletics (4, 24, 30). Suggesting care for all athletes is paramount for their mental and physical health. Within Division I, when surveying athletic trainers (health-care providers to athletes), they believe better care would be more readily available to student-athletes if mental health services occurred onsite in the Athletic Training Facility. However, only 43% of head athletic trainers used screening instruments to assess for mental health disorders (36). Due to the differences in resources, Division III athletics does not have the same degree of access to physicians, yet alone mental health professionals with a background in sports or college athletics (37). Substance use has demonstrated negative impacts on both academic and athletic success with more than 25% missing class and 16% performing poorly on a test or in practice/games due to use (16). Student-athletes tend to use substances to help with depression and anxiety, however, substance use also increase negative symptoms regarding mental health. Congruent tools and screening for mental health and substance use are paramount for quick identification and intervention to those that have elevated scores.

### Limitations and future research

There were limitations identified in this study. First, all data was self-reported measurements and relied on honest answers to the best of the participant's ability. This was mitigated through the selection of four validated measures specifically for anxiety, depression, alcohol use and substance use. They are used as screening tools to indicate whether there is a need for follow-up to then further diagnose a patient, not used for diagnosis. Another limitation of this study was that despite meeting saturation for power, the overall demographic of our sample size is not representative of a whole collective of 18–25-year-old student-athletes across all levels of NCAA competition and sex was not distributed evenly between males and females nor represented despite recruitment efforts via social media. A major limitation regarding follow-up care was due to the anonymous nature of the survey. Researchers could not follow-up with the participants expressing self-harm thoughts or other concerns they may have had in participating in the research study. With IRB approval, worldwide mental health resources were listed at the beginning and end of the survey to provide resources for those that may have wanted to seek help or if certain questions made individuals feel uncomfortable or distressed.

As more openness to discussing mental health in athletics grows, future research should start to focus on more validated measures that identify mental health (anxiety and depression) and substance use specifically in the student-athlete population and identifying what risk factors more likely dispose a student-athlete to increased mental health distress and substance use including the Sport Mental Health Assessment Tool 1 (SMHAT-1) and Sport Mental Health Recognition Tool 1 (SMHRT-1) by the International Olympic Committee (38). In the new and changing world of college and high school athletics factors like specific sports, social media engagement, NIL, longer sports seasons, and the transfer portal creates more concerns for the toll on mental health and risk of substance use of the student-athletes. Firstly, identify the causation of increased anxiety and depression symptoms in this new athletic environment, then target those areas with effective interventions that can help prevent, reduce, or alleviate the student-athletes’ symptoms. Finally, an area that is underserved includes the youth athletes (middle school to high school). There is less organized care at these levels and is an underserved population that are dealing with similar stressors (academics, athletics, social media, etc.) as collegiate athletes with less resources available to them. Some screening tools are transferable to that age range, however research for patients younger than 18 years old include adolescent tools can be used for assessment of substance use: Alcohol screening and brief intervention for youth (9–18yrs) and the CRAFFT (car, relax, alone, forget, friends, and trouble): identifies substance use/risk for those 12–21yrs.

## Conclusions

This study identified that most participants experienced elevated symptoms of anxiety and depression, as well as harmful risk of substance use disorders. Relationships were discovered between anxiety, depression, substance use, age, sex, ethnicity, competition level, athletic year, and current season of sport. These results highlight the increased prevalence of anxiety, depression and substance use symptoms among student-athletes, specifically providing insight into an understudied demographic at the Division III level. Mental health is just as important as physical health in student-athletes. Mental health issues often lead to subsequent substance use. While regular college students may participate in the use of substances, student-athletes can have more intense uses of substances, which can lead to substance use disorders and addiction. This affects the student-athlete's life academically, athletically, physically, and continues to more intense mental health issues. Clinicians need to focus more efforts on early screening tools to identify those at higher risk for anxiety, depression and substance use symptoms. Overall, clinicians need to have confidence in utilizing screening tools and competence in recognizing mental health problems and substance use symptoms in student-athletes to make impactful referrals when needed.

## Data Availability

The raw data supporting the conclusions of this article will be made available by the authors, without undue reservation.
